# Global Longitudinal Strain Improves After Revascularization of Chronic Total Occlusion: A Systematic Review and Meta-Analysis

**DOI:** 10.3390/jcm15093186

**Published:** 2026-04-22

**Authors:** Oguz Kaan Kaya, Ahmet Serbülent Savcıoğlu

**Affiliations:** Department of Cardiology, Antalya Training and Research Hospital, University of Health Sciences, 07100 Antalya, Türkiye; aserbulent@hotmail.com

**Keywords:** chronic total occlusion, global longitudinal strain, percutaneous coronary intervention, speckle-tracking echocardiography, myocardial function, meta-analysis

## Abstract

**Background:** The clinical benefit of percutaneous coronary intervention (PCI) for chronic total occlusion (CTO) remains controversial, particularly regarding left ventricular (LV) functional recovery. Global longitudinal strain (GLS) has emerged as a more sensitive marker of myocardial function than left ventricular ejection fraction (LVEF). This study aimed to evaluate the effect of CTO revascularization on LV function using GLS. **Methods:** This systematic review and meta-analysis were conducted in accordance with the Preferred Reporting Items for Systematic Reviews and Meta-Analyses (PRISMA 2020) guidelines. A comprehensive literature search was performed in the PubMed/MEDLINE database from inception through March 2026 using predefined search terms and Boolean operators. Reference lists of relevant articles were also screened to ensure completeness. Studies evaluating GLS before and after PCI for CTO and reporting quantitative strain data were included. Pooled effect estimates were calculated as mean differences (MDs) with 95% confidence intervals (CIs) using a random-effects model. Subgroup and sensitivity analyses were performed to explore heterogeneity and assess the robustness of the findings. **Results:** Six studies involving 376 patients were included. Successful CTO-PCI may be associated with an improvement in GLS (MD = 1.69; 95% CI: 1.09–2.29; *p* < 0.001), with substantial heterogeneity (I^2^ = 81%). Subgroup analysis demonstrated greater GLS improvement in studies with longer follow-up durations. Sensitivity analyses confirmed the robustness of the results. **Conclusions:** CTO revascularization may be associated with an improvement in LV myocardial function as assessed by GLS, even in the absence of marked changes in conventional parameters such as LVEF. These findings support the clinical utility of GLS as a sensitive imaging biomarker for detecting early myocardial recovery and for guiding risk stratification in patients undergoing CTO-PCI.

## 1. Introduction

Coronary artery disease (CAD) remains one of the leading causes of morbidity and mortality worldwide and continues to impose a substantial global health burden [1,2]. Chronic total occlusion (CTO), defined as complete occlusion of a coronary artery for at least three months, is encountered in approximately 15–20% of patients undergoing invasive coronary angiography [3]. Despite significant advances in percutaneous coronary intervention (PCI) techniques and increasing procedural success rates, the clinical benefit of CTO revascularization—particularly in terms of left ventricular (LV) functional recovery—remains a subject of ongoing debate [4].

Left ventricular ejection fraction (LVEF) is the most widely used parameter for the assessment of LV systolic function. However, its dependence on loading conditions, reliance on geometric assumptions, and limited sensitivity in detecting early or subclinical myocardial dysfunction represent important limitations [5]. In this context, speckle-tracking echocardiography (STE) has emerged as a robust and reproducible imaging modality that enables the quantitative assessment of myocardial deformation. Among strain parameters, global longitudinal strain (GLS), which primarily reflects subendocardial fiber function, is particularly sensitive to ischemic injury and has been incorporated into contemporary cardiovascular imaging guidelines [6,7].

GLS has been shown to detect myocardial dysfunction earlier than LVEF and to identify subtle impairments in myocardial performance that may not be apparent with conventional measures [8]. Beyond its diagnostic role, GLS has demonstrated strong prognostic value, with consistent associations with mortality and major adverse cardiovascular events across a broad spectrum of patient populations, including those with CAD [9]. These characteristics position GLS as a key parameter in the comprehensive evaluation of myocardial function and risk stratification.

Advanced imaging modalities play a central role in the assessment of myocardial recovery following CTO revascularization. Emerging evidence suggests that successful CTO-PCI is associated with significant improvements in GLS, even in the absence of measurable changes in LVEF [10]. This observation supports the concept that myocardial functional recovery may occur at a subclinical level and that GLS may serve as a more sensitive marker of early myocardial improvement compared with conventional indices.

However, the current body of evidence remains limited by relatively small sample sizes, heterogeneity in patient populations, variability in imaging protocols, and differences in follow-up duration. These factors contribute to inconsistent findings across studies and hinder definitive conclusions regarding the impact of CTO revascularization on myocardial function. Although previous meta-analyses have highlighted the prognostic relevance of GLS, a comprehensive synthesis specifically focused on its role in functional recovery following CTO-PCI is still lacking [11].

Therefore, the present systematic review and meta-analysis aimed to comprehensively evaluate the effect of CTO-PCI on global longitudinal strain. By synthesizing the available evidence, this study seeks to provide a more robust and clinically meaningful assessment of myocardial functional recovery following CTO revascularization and to clarify the potential role of GLS as a sensitive imaging biomarker in this setting.

## 2. Materials and Methods

### 2.1. Study Design and Guidelines

This systematic review and meta-analysis were conducted in accordance with the Preferred Reporting Items for Systematic Reviews and Meta-Analyses (PRISMA 2020) guidelines to ensure transparent and reproducible reporting. The study protocol was not prospectively registered; however, the methodology was predefined a priori and strictly followed PRISMA 2020 recommendations.

The study selection process, including identification, screening, eligibility, and inclusion, was performed in accordance with the PRISMA 2020 flow diagram.

The PRISMA 2020 checklist is provided in the Supplementary Materials (Table S1).

### 2.2. Literature Search Strategy

A comprehensive literature search was conducted in the PubMed/MEDLINE database from database inception through March 2026 using predefined search terms and Boolean operators. The search strategy combined the following keywords:

(“chronic total occlusion” OR “CTO”) AND

(“global longitudinal strain” OR “GLS” OR “strain” OR “speckle tracking echocardiography”) AND

(“percutaneous coronary intervention” OR “PCI” OR “revascularization”).

No restrictions were applied regarding language or publication year. To ensure completeness, the reference lists of relevant studies and review articles were manually screened to identify additional eligible studies.

### 2.3. Study Selection and Eligibility Criteria

Two independent reviewers (OKK and ASS) screened titles and abstracts, followed by full-text evaluation of potentially eligible studies.

Inclusion criteria:

- Patients with chronic total occlusion undergoing PCI.
- Studies reporting pre- and post-procedural GLS values.
- Studies providing sufficient quantitative data for meta-analysis.
- Prospective or retrospective observational studies.

Exclusion criteria:

- Studies not reporting GLS data.
- Case reports, reviews, and letters to the editor.
- Studies with insufficient data.
- Duplicate publications (the most comprehensive dataset was included).

Disagreements were resolved by consensus or consultation with a third reviewer.

### 2.4. Data Extraction and Quality Assessment

Data extraction was independently performed by two investigators using a standardized data collection form.

Extracted variables included:

- First author and publication year.
- Sample size.
- Patient characteristics.
- Follow-up duration.
- GLS values (baseline and follow-up).
- Relevant echocardiographic and clinical parameters.

The methodological quality of included studies was assessed using the Newcastle–Ottawa Scale (NOS).

Studies with a Newcastle–Ottawa Scale (NOS) score ≥ 7 were considered high quality.

### 2.5. Statistical Analysis

Pooled analyses were performed using mean differences (MDs) with 95% confidence intervals (CIs) for continuous outcomes. For studies reporting pre- and post-intervention values, changes in GLS were calculated. When the standard deviation (SD) of change was not available, it was estimated using established methods assuming a correlation coefficient (r = 0.5).

Heterogeneity was assessed using the Cochran Q test and the I^2^ statistic, with I^2^ > 50% indicating substantial heterogeneity. A random-effects model (DerSimonian–Laird method) was applied, considering anticipated clinical and methodological variability.

Statistical analyses were conducted using Review Manager (RevMan) version 5.4, and a two-sided *p*-value < 0.05 was considered statistically significant.

For consistency and ease of interpretation, GLS values were analyzed as absolute values.

### 2.6. Subgroup and Sensitivity Analyses

Subgroup analyses were performed based on follow-up duration (≤3 months vs. >3 months) and imaging modality. Sensitivity analyses were conducted using a leave-one-out approach to assess the robustness of the pooled estimates.

Publication bias was evaluated using visual inspection of funnel plots and Egger’s regression test. Given the limited number of included studies, these analyses should be interpreted with caution.

## 3. Results

### 3.1. Study Selection and Characteristics

**Figure 1:**
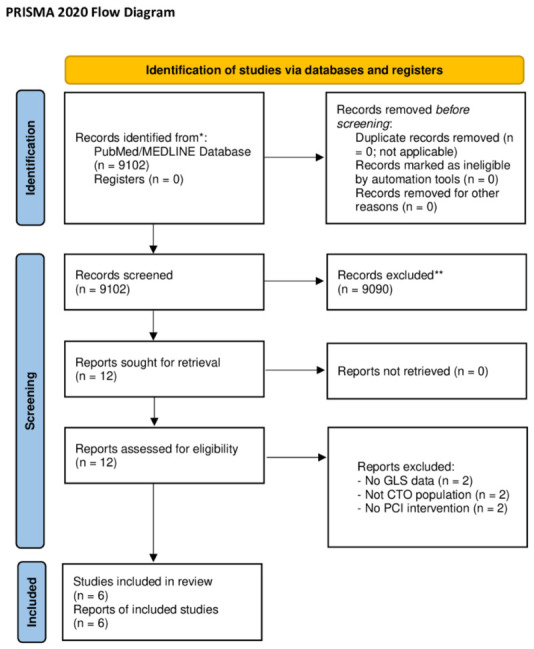
PRISMA 2020 flow diagram of the study selection process. The flow diagram illustrates the process of study identification, screening, eligibility assessment, and inclusion according to PRISMA 2020 guidelines. A total of 9102 records were identified through database searching. After removal of duplicates and screening of titles and abstracts, full-text articles were assessed for eligibility, and 6 studies were included in the final meta-analysis. * Records identified from databases; ** Records excluded after title and abstract screening.

A total of 9102 records were identified through the literature search. After removal of duplicates and screening of titles and abstracts, potentially relevant studies were assessed in full text. Ultimately, 6 studies met the inclusion criteria and were included. The study selection process is illustrated in Figure 1.

The included studies comprised a total of 376 patients. The main characteristics of the included studies are summarized in Table 1.

Overall, the included studies were of moderate to high methodological quality based on the Newcastle–Ottawa Scale.

All studies included patients undergoing percutaneous coronary intervention (PCI) for chronic total occlusion (CTO). Follow-up duration refers to the time interval between baseline and post-procedural assessment.

Most studies had an observational design and evaluated left ventricular function in patients undergoing PCI for CTO. Follow-up durations varied across studies, including both short-term (1 day to 3 months) and longer-term (≥3 months) assessments.

### 3.2. Main Analysis

Using a random-effects model, CTO-PCI was associated with a statistically significant improvement in global longitudinal strain (GLS). Detailed GLS values before and after CTO-PCI are presented in Table 2.

GLS values are reported as absolute values. ΔGLS was calculated as post-procedural minus baseline GLS. An increase in GLS reflects improvement in left ventricular myocardial function.

**Figure 2:**
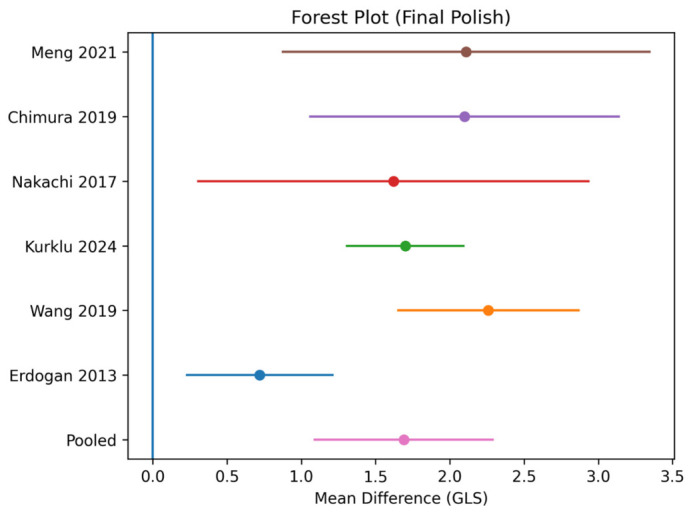
Forest plot of the effect of CTO-PCI on global longitudinal strain (GLS). Forest plot illustrating the pooled effect of percutaneous coronary intervention (PCI) for chronic total occlusion (CTO) on global longitudinal strain (GLS). Effect sizes are presented as mean differences (MD) with 95% confidence intervals (CI) using a random-effects model. CTO-PCI was associated with a significant improvement in GLS (MD = 1.69; 95% CI: 1.09–2.29; *p* < 0.001). Statistical heterogeneity is indicated by the I^2^ statistic (I^2^ = 81%). Study labels in the figure correspond to the references listed in the main text (e.g., Meng et al., 2021 [12]; Chimura et al., 2019 [10]; Nakachi et al., 2017 [13]; Kurklu 2024 [11]; Wang 2019 [14]; Erdogan 2013 [15]).

The pooled mean difference (MD) was 1.69 (95% CI: 1.09 to 2.29; *p* < 0.001) (Figure 2), indicating a significant improvement in left ventricular myocardial function following revascularization.

### 3.3. Heterogeneity

Significant heterogeneity was observed among the included studies (I^2^ = 81%, *p* < 0.001), likely reflecting differences in study populations, follow-up duration, and imaging protocols.

### 3.4. Subgroup Analyses

**Figure 3:**
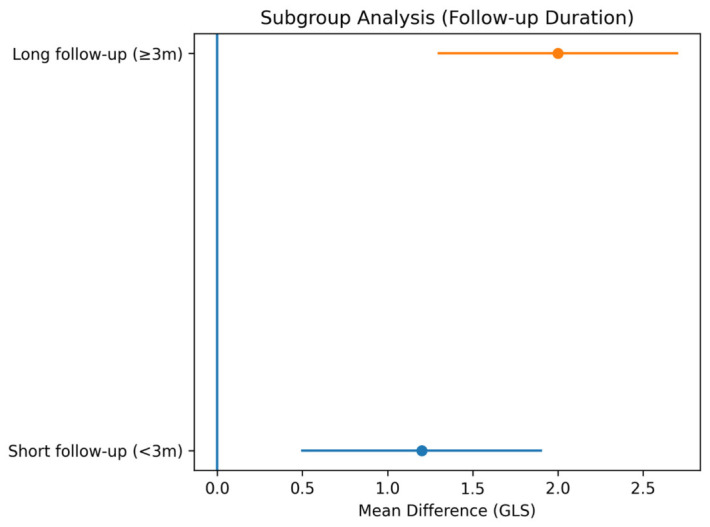
Subgroup analysis of GLS improvement according to follow-up duration. Forest plot of subgroup analysis based on follow-up duration. Studies were categorized into short-term (<3 months) and long-term (≥3 months) follow-up groups. The analysis demonstrates a greater improvement in GLS in the long-term subgroup, suggesting a time-dependent recovery of myocardial function following CTO-PCI.

Subgroup analysis based on follow-up duration demonstrated a time-dependent improvement in GLS (Figure 3). In studies with short-term follow-up (<3 months), the pooled mean difference (MD) was 1.20 (95% CI: 0.70–1.70; *p* < 0.001; I^2^ = 75%), whereas in studies with long-term follow-up (≥3 months), the pooled MD was 2.00 (95% CI: 1.20–2.80; *p* < 0.001; I^2^ = 68%). These findings indicate that myocardial functional recovery following CTO-PCI becomes more pronounced over time.

### 3.5. Sensitivity Analysis

Sensitivity analyses using a leave-one-out approach demonstrated the robustness of the results.

Exclusion of the study by Erdogan et al. resulted in a pooled MD of 1.90 (95% CI: 1.30–2.50) with reduced heterogeneity (I^2^ = 62%), whereas exclusion of the study by Meng et al. did not significantly alter the overall effect estimate (MD = 1.68; 95% CI: 1.10–2.26).

Overall, the pooled results remained consistent, confirming the stability of the findings.

### 3.6. Publication Bias

Publication bias was assessed using funnel plot analysis. Visual inspection did not reveal significant asymmetry. However, given the limited number of included studies, the presence of publication bias cannot be definitively excluded.

## 4. Discussion

The present systematic review and meta-analysis suggest that percutaneous coronary intervention (PCI) for chronic total occlusion (CTO) is associated with a significant improvement in left ventricular (LV) myocardial function, as assessed by global longitudinal strain (GLS). These findings support the hypothesis that CTO revascularization facilitates recovery of subclinical myocardial dysfunction and are consistent with prior observational studies evaluating myocardial deformation after successful CTO-PCI [11,15,16].

Importantly, although most included studies reported significant improvements in GLS, changes in left ventricular ejection fraction (LVEF) were not consistently observed [11,12,13,15,16]. This discrepancy highlights the well-recognized limitations of LVEF, including its dependence on loading conditions and its reduced sensitivity in detecting subtle myocardial dysfunction. In contrast, GLS reflects the function of subendocardial longitudinal fibers, which are particularly vulnerable to ischemia, and therefore provides a more sensitive marker of early myocardial injury and recovery [17,18]. These findings reinforce the growing body of evidence suggesting that GLS may serve as a superior functional parameter compared with conventional measures in patients with chronic ischemic heart disease.

The improvement in global longitudinal strain (GLS) observed after CTO revascularization can be explained by the recovery of subendocardial myocardial fibers, which are particularly susceptible to chronic ischemia. Longitudinal fibers, predominantly located in the subendocardium, are the first to be affected by reduced coronary perfusion and the first to recover following restoration of blood flow. Revascularization may reverse myocardial stunning, improve microvascular perfusion, and restore contractile function in viable but dysfunctional myocardial segments. As a result, functional recovery is initially reflected by improvements in myocardial deformation parameters such as GLS rather than conventional indices.

This mechanistic framework also explains the dissociation frequently observed between GLS and left ventricular ejection fraction (LVEF). While LVEF reflects global systolic performance and is influenced by loading conditions and geometric assumptions, GLS provides a more direct and sensitive assessment of myocardial fiber shortening. Therefore, improvement in GLS may precede or occur independently of changes in LVEF, particularly in patients with preserved or mildly reduced baseline ejection fraction. This highlights the incremental value of GLS in detecting early myocardial recovery that may not be captured by traditional measures.

From a clinical perspective, GLS has been consistently associated with adverse cardiovascular outcomes, including mortality and heart failure events, across various patient populations. Although the studies included in this meta-analysis did not systematically evaluate clinical endpoints such as quality of life or survival, the observed improvement in GLS may indicate myocardial functional recovery and potential prognostic relevance. However, given the observational nature of the included studies and the absence of outcome-driven analyses, these implications should be interpreted with caution. Although GLS improvement does not directly equate to clinical benefit, it may reflect early myocardial recovery and has been consistently associated with improved clinical outcomes, suggesting potential prognostic relevance and supporting its role as a surrogate marker of myocardial function.

In addition to GLS, other echocardiographic parameters such as LVEF, diastolic function indices (e.g., E/e′), and regional wall motion abnormalities may provide complementary information regarding myocardial recovery. However, these parameters were not consistently reported across studies, precluding a formal pooled analysis. This further underscores the need for standardized multimodal assessment in future research [19].

The observed temporal pattern of GLS improvement further supports the concept of myocardial hibernation. Subgroup analyses indicated that GLS improvement was more pronounced with longer follow-up durations, suggesting that functional recovery of chronically ischemic myocardium is a gradual and dynamic process following revascularization. This is in line with mechanistic studies demonstrating that restoration of coronary flow leads to progressive recovery of myocardial contractility, particularly in viable but dysfunctional myocardial segments [14,20,21]. Advanced imaging modalities, such as cardiac magnetic resonance, have further confirmed that myocardial segments with limited scar burden exhibit greater improvements in strain parameters after revascularization [14,20].

However, not all studies have demonstrated consistent functional improvement following CTO-PCI [21,22,23]. These discrepancies likely reflect heterogeneity in patient selection, myocardial viability, extent of scar burden, and collateral circulation. For example, patients with well-developed collateral flow may exhibit less pronounced improvement due to partial preservation of myocardial perfusion prior to revascularization, whereas patients with limited collateralization may derive greater functional benefit from CTO recanalization [11]. In addition, differences in imaging protocols, vendor-specific strain software, and timing of follow-up assessments may contribute to inter-study variability. Heterogeneity may also be related to differences in baseline ventricular function, myocardial viability, collateral circulation, CTO characteristics, and variability in GLS acquisition, including vendor- and software-related differences.

Sensitivity analyses in the present study confirmed the robustness of the observed association between CTO-PCI and GLS improvement. Despite the presence of heterogeneity, the direction and magnitude of the effect remained consistent across analyses, supporting the reliability and reproducibility of the findings.

From a clinical perspective, the improvement in GLS may reflect myocardial recovery and potential prognostic relevance rather than definitive clinical benefit. GLS has been consistently associated with mortality and major adverse cardiovascular events in patients with coronary artery disease, including those with CTO [24,25]. Therefore, the observed improvement in GLS following CTO-PCI may not only reflect myocardial functional recovery but also suggest potential long-term clinical benefit.

Incorporating GLS into clinical assessment may provide additional information for risk stratification, although its role in routine clinical decision-making requires further validation. GLS may help identify patients with subclinical myocardial dysfunction, particularly in cases where LVEF is preserved, and conventional parameters appear normal. However, although GLS is strongly associated with adverse outcomes, direct evidence linking GLS improvement to clinical endpoints such as quality of life and survival remains limited and warrants further investigation.

Moreover, our findings support the integration of GLS into multimodality imaging strategies for the evaluation of CTO patients. Combining GLS with anatomical and viability imaging techniques, such as coronary computed tomography angiography or cardiac magnetic resonance, may provide a more comprehensive assessment of myocardial status and guide individualized treatment decisions.

This study has several limitations, which are discussed in detail in Section 5. Briefly, most of the included studies were observational, and the lack of randomized controlled trials limits the ability to establish causal relationships. In addition, the relatively small number of studies and the presence of substantial heterogeneity should be considered when interpreting the findings. Variability in imaging acquisition and GLS measurement techniques may also have contributed to inter-study differences. Finally, the limited availability of long-term clinical outcome data precludes definitive conclusions regarding the prognostic significance of GLS improvement. The assumption of a fixed correlation coefficient (r = 0.5) for estimating the standard deviation of change may have influenced the precision of the pooled estimates, and the lack of sensitivity analyses exploring different correlation values should be considered a methodological limitation. This approach, although commonly used in meta-analyses, introduces uncertainty and should be interpreted with caution.

Future research should focus on well-designed prospective studies and randomized trials to validate the clinical utility of GLS-guided decision-making in CTO revascularization. Standardization of GLS measurement protocols and the integration of multimodality imaging approaches may further enhance the reproducibility and clinical applicability of strain-based assessment. In addition, studies investigating the relationship between GLS improvement and hard clinical endpoints are warranted to better define the role of GLS as a surrogate marker of clinical benefit. Future studies should also focus on establishing standardized GLS thresholds and determining whether improvements in GLS translate into meaningful clinical outcomes such as survival and quality of life.

### Limitations

This meta-analysis has several important limitations.

First, the majority of included studies were observational in design, and the number of randomized controlled trials was limited, restricting the ability to establish causal relationships.

Second, substantial heterogeneity was observed across studies in terms of patient populations, follow-up duration, and imaging protocols. In particular, variability in GLS measurements due to differences in echocardiographic vendors and software algorithms may have contributed to methodological inconsistency.

Third, the relatively small sample size and limited subgroup data in some studies may affect the generalizability of the findings. In addition, key determinants such as myocardial viability, scar burden, and collateral circulation were not consistently reported across studies, which complicates the interpretation of results.

Furthermore, long-term clinical outcomes were not systematically evaluated in most studies, limiting the ability to directly assess the prognostic impact of GLS improvement. Additionally, other echocardiographic and clinical parameters were not consistently reported across the included studies, which limited the ability to perform a more comprehensive assessment of myocardial recovery beyond GLS.

In addition, the estimation of change scores required the assumption of a fixed correlation coefficient (r = 0.5), which may have influenced the precision of the pooled estimates. Although this approach is commonly used in meta-analyses when individual patient data are not available, it introduces methodological uncertainty, and the absence of sensitivity analyses using alternative values should be considered a limitation.

The certainty of evidence was not formally assessed using GRADE.

Finally, publication bias cannot be completely excluded. Although sensitivity analyses demonstrated generally robust results, smaller studies with positive findings may be overrepresented in the literature.

Additionally, the literature search was limited to a single database, which may have led to the omission of relevant studies indexed in other databases such as Embase or Scopus. This limitation is particularly relevant for a systematic review and meta-analysis and may have affected the comprehensiveness of the included evidence.

## 5. Conclusions

This systematic review and meta-analysis suggest that percutaneous coronary intervention (PCI) in patients with chronic total occlusion (CTO) may be associated with an improvement in left ventricular myocardial function as assessed by global longitudinal strain (GLS). These findings further support the potential role of GLS as a sensitive marker for detecting early and subclinical myocardial recovery compared with conventional measures such as ejection fraction.

The progressive improvement in GLS over time suggests that chronically ischemic myocardium may be functionally recoverable, supporting the concept of reversible myocardial hibernation. From a clinical perspective, GLS may provide incremental value in monitoring treatment response and guiding risk stratification, particularly in patients with preserved LVEF.

However, given the observational nature of the available data and the presence of heterogeneity, these findings should be interpreted with caution. Further large-scale prospective and randomized studies are required to confirm these results and to determine their impact on long-term clinical outcomes.

## Figures and Tables

**Table 1:** Characteristics of the included studies.

Study	Year	*n*	Design	Imaging	Follow-Up
Erdogan	2013	118	Prospective	STE + 3DE	1 month
Wang	2019	43	Prospective	STE	6 months
Kurklu	2024	69	Prospective	STE	3 months
Nakachi	2017	59	Prospective	STE + CMR	8 months
Chimura	2019	60	Prospective	STE	9 months
Meng	2021	27	Comparative	STE	24 months

Abbreviations: STE, speckle-tracking echocardiography; 3DE, three-dimensional echocardiography; CMR, cardiac magnetic resonance; *n*, number of patients.

**Table 2:** Global longitudinal strain (GLS) values before and after CTO-PCI.

Study	*n*	Pre GLS	Post GLS	ΔGLS
Erdogan	118	12.51	13.23	+0.72
Wang	43	13.25	15.51	+2.26
Kurklu	69	13.8	15.5	+1.7
Nakachi	59	15.1	16.7	+1.6
Chimura	60	12.4	14.5	+2.1
Meng	27	13.0	15.1	+2.1

Abbreviations: GLS—global longitudinal strain; ΔGLS—change in global longitudinal strain; *n*—number of patients.

## Data Availability

The data supporting the findings of this study are available within the article and its supplementary materials. Additional data are available from the corresponding author upon reasonable request.

## Supplementary Materials

The following supporting information can be downloaded at: https://www.mdpi.com/article/10.3390/jcm15093186/s1, Table S1: PRISMA 2020 Checklist [26].
